# Enhancing green supplier selection: A nonlinear programming method with TOPSIS in cubic Pythagorean fuzzy contexts

**DOI:** 10.1371/journal.pone.0310956

**Published:** 2024-12-05

**Authors:** Musa Khan, Wu Chao, Muhammad Rahim, Fazli Amin

**Affiliations:** 1 School of Finance and Economics, Jiangsu University, Zhenjiang, Jiangsu, P. R. China; 2 Department of Mathematics and Statistics, Hazara University, Mansehra, Khyber Pakhtunkhwa, Pakistan; National Kaohsiung University of Science and Technology, TAIWAN

## Abstract

The advancements in information and communication technologies have given rise to innovative developments such as cloud computing, the Internet of Things, big data analytics, and artificial intelligence. These technologies have been integrated into production systems, transforming them into intelligent systems and significantly impacting the supplier selection process. In recent years, the integration of these cutting-edge technologies with traditional and environmentally conscious criteria has gained considerable attention in supplier selection. This paper introduces a novel Nonlinear Programming (NLP) approach that utilizes the Technique for Order Preference by Similarity to Ideal Solution (TOPSIS) method to identify the most suitable green supplier within cubic Pythagorean fuzzy (CPF) environments. Unlike existing methods that use either interval-valued PFS (IVPFS) or Pythagorean fuzzy sets (PFS) to represent information, our approach employs cubic Pythagorean fuzzy sets (CPFS), effectively addressing both IVPFS and PFS simultaneously. The proposed NLP models leverage interval weights, relative closeness coefficients (RCC), and weighted distance measurements to tackle complex decision-making problems. To illustrate the accuracy and effectiveness of the proposed selection methodology, we present a real-world case study related to green supplier selection.

## 1. Introduction

The concept of ’green’ is a vital paradigm in supply chain management, functioning as an organizational philosophy. The importance of green supply chain management (GSCM) has grown substantially due to stricter environmental regulations and increasing consumer demand for sustainability [1]. GSCM is a comprehensive management approach that incorporates environmental considerations into all supply chain activities, from product design and material selection to purchasing and production processes. By adopting GSCM practices, companies can enhance both their environmental performance and profitability by reducing the impact of environmental risks [2]. For GSCM to be effective, it must be implemented across every stage of the supply chain, beginning with raw material procurement and continuing through to the recycling or disposal of the final product. Focusing solely on green practices in inbound supply chain operations is inadequate for meeting environmental objectives. Companies must also address the environmental impact of outbound operations, engaging their partners and stakeholders to enhance supplier performance [3]. As a result, suppliers are pivotal in driving environmental improvements within companies [4]. Consequently, the selection of green suppliers (GSS) has become a critical element in the successful implementation of GSCM practices.

The green concept, which is a crucial paradigm in supply chain management, can be viewed as an organizational philosophy. The prominence of the green supply chain management (GSCM) concept has increased significantly in response to environmental regulations and growing consumer demands for sustainability [1]. GSCM represents a management approach that integrates environmental considerations into all supply chain operations, including product design, material selection, purchasing, and production processes. By implementing GSCM practices, companies can enhance their environmental performance and profitability by minimizing the impact of environmental risks [2]. GSCM must be implemented at every stage of the supply chain, starting with the procurement of raw materials and extending to the recycling or disposal of the final product. Simply focusing on green practices at the inbound supply chain operations is insufficient for achieving environmental goals and solutions. To improve the performance of their suppliers, companies must also address the environmental impacts of outbound operations among their partners and stakeholders [3]. Therefore, suppliers play a crucial role in facilitating environmental improvements for companies [4]. Consequently, while implementing GSCM practices, companies have recognized the significance of green supplier selection (GSS) in the supply chain.

Multi-criteria decision-making (MCDM) approaches can serve as effective tools for addressing the supplier selection (GSS) problem and facilitating supplier comparison. While there exist several studies on GSS in the literature, conducted under both crisp and fuzzy environments. For example, Zulqarnain et al. [5] introduced a TOPSIS method based on correlation coefficients within a PF soft environment and demonstrated its application in green supply chain management. Ahmad et al. [6] explored the use of the Pythagorean fuzzy soft Einstein ordered weighted average operator in addressing sustainable supplier selection problems. Cenk et al. [7] introduced a Pythagorean fuzzy TOPSIS method for selecting green suppliers in the food industry. further research is required to explore new criteria, incorporate diverse areas of expertise and linguistic variables. Such research can help in developing more sophisticated and robust decision-making models that can handle the complexities and uncertainties inherent in modern supply chain management systems. By leveraging MCDM techniques and advanced technologies, organizations can improve their supplier selection processes and gain a competitive edge in the marketplace.

### 1.1. A systematic literature review on TOPSIS and CPFSs for MCDM

Information measures offer alternative approaches to combining different characteristics of objects that have been extensively studied. These measures provide a way to quantify and compare the amount of information contained in different sets of data or variables. By using information measures, researchers can gain a deeper understanding of the underlying patterns and relationships within the data, and make more informed decisions based on this knowledge. Under it, a TOPSIS technique [8] is a famous procedure in which the optimal solution is designated based on the smallest distance from its mention sets. The benefits of the TOPSIS technique are that it considers the distance between the sets and their similarities or dissimilarity to avoid drawing decisions based on a tiny distance. Different strategies have been developed by researchers employing IFS, IVIFS, PFS, and IVPFS to handle the MCDM problems by adopting these advantages of TOPSIS techniques. For instance, Hung and Chen [9] used Shannon’s entropy to handle the MCDM issue using the TOPSIS technique. Park et al. [10] introduced the TOPSIS technique for solving MCDM issues using IVIFSs. Izadikhah [11] used the TOPSIS technique to investigate supplier selection challenges in the IVIFS context. Li [12] provided an NPL model for calculating the limit of the TOPSIS approach’s relative closeness coefficients (RCCs) in the IVIFS context. Zhang and Xu improved the TOPSIS technique for dealing with MCDM with PFS. Ak and Gul [13] presented a risk analysis approach based on the analytic hierarchy process TOPSIS integration upgraded with PFS and applied to the information security hierarchy process. Oz et al. [14] proposed a method for assessing occupational health and safety risks using an extended TOPSIS with PFS. Yu et al. [15] presented a group DM sustainable supplier selection technique in the IVPFS context by extending TOPSIS. Lin et al. [16] developed the linguistic Pythagorean fuzzy TOPSIS approach for dealing with DM situations. Garg [17] proposed sine trigonometric operational laws and their AOs under Pythagorean fuzzy environment to solve MCDM problems. Jana et al. [18] presented Dombi AOs and their applications in MCDM problems. Garg and Deng [19] presented the idea of Archimedean Bonferroni mean operators under complex PF information to handle the complex environment in the DM process. Amin et al. [20] proposed the generalized series of AOs under CPFSs. Rahim et al. [21] proposed a series of Bonferroni mean AOs to solve MCDM problems. Zulqarnain et al. [22] developed a TOPSIS technique within a PF hypersoft environment based on correlation coefficients, applying it to the selection of antivirus masks during the COVID-19 pandemic. Zulqarnain et al. [23] extended the correlation coefficient-based TOPSIS technique for interval-valued Pythagorean fuzzy soft sets, applying it in a case study focused on extract, transform, and load (ETL) techniques. For additional related studies, readers can refer to references [24,25].

Although the scenarios contribute significantly to hesitations in termination, there are circumstances in which the evaluator must estimate the falsehood associated with the accuracy value fluctuating throughout an interval. To address this issue, Jun et al. [26] proposed the concept of cubic sets (CS), which are considered appropriate tools for handling any discrepancies between agreed interval values and vice versa. In addition, Vijayabalaji and Sivaramakrishnan [27] defined certain concepts of cubic linear spaces, such as "P-union and R-union," as well as their corresponding internal and external cubic linear spaces. The theory of CS, sub-algebras, ideals, and the presentation of the closed ideals of B-algebras [28] were also developed. Mahmood et al. [29] combined hesitant data with CS to create the cubic hesitant fuzzy set (FS). Based on CS, Kang and Kim [30] explored the concept of images and inverse of CS. However, since CS cannot account for the non-monotonicity degree (NMD) that corresponds to decision-making (DM), their theory is limited in nature. To manage fuzzy information, Kaur and Garg [31,32] introduced the concept of cubic interval-valued fuzzy sets (CIFS) by combining the structure of interval-valued fuzzy sets (IVFS) and IFS. Each CIFS element is represented as $\left(\langle \left[U^{-},U^{+}],[V^{-},V^{+}\right]\rangle ,\langle U,V\rangle \right)$ fulfilling the requirements *ξ*^+^+*ϕ*^+^≼1 and *ξ*+*ϕ*≼1. However, in some real cases, $U^{+}+V^{+}≼1$ or U+U≼1 cannot be met. As a result, Abbas et al. [33] proposed the concept of the cubic picture fuzzy CPFS, which is a generalization of CIFS. Each CPFS element satisfies the conditions ${\left(U^{+}\right)}^{2}+{\left(V^{+}\right)}^{2}≼1$ and ${\left(U\right)}^{2}+{\left(V\right)}^{2}≼1$ and is expressed as $\left(\langle \left[U^{-},U^{+}],[V^{-},V^{+}\right]\rangle ,\langle U,V\rangle \right)$. For instance, consider the following scenario: an investor wants to invest in the domestic market and needs to determine the estimated importance of his investment before participating. He realized that it would be 75-80% in favor of profit and 40-50% in favor of loss. However, after a few months, he realized that his result was 60% consistent with his prior profit assessment while it was 45% inconsistent with his loss assessment. The P-order CPFS representation of such information is (〈[0.70,0.80],[0.40,0.50]〉,〈0.60,0.45〉). Furthermore, if instead of 60% and 45%, the individual finds that 45% are inconsistent with the profit projections and 65% are consistent with the loss predictions, the result is (〈[0.70,0.80],[0.40,0.50]〉,〈0.65,0.45〉), which is referred to as R-order CPFS. These are not available for CIFS, as the sum of 0.8 and 0.6 is greater than 1. However, they are available for CPFS as 0.80^2^+0.60^2^≼1.

### 1.2. Green supplier selection

Green supplier selection is the process of evaluating and selecting suppliers based on their environmental performance and sustainability practices. This can include considerations such as the supplier’s energy and resource efficiency, waste reduction efforts, and compliance with environmental regulations. The goal of green supplier selection is to minimize the environmental impact of a company’s supply chain and to support the adoption of sustainable practices throughout the industry. In the green supplier selection process, a company may consider a variety of factors, such as the supplier’s greenhouse gas emissions, water usage, and waste generation. The company may also evaluate the supplier’s environmental policies and initiatives, as well as its track record of environmental compliance. By choosing suppliers that are environmentally responsible, a company can reduce its own environmental footprint and support the transition to a more sustainable economy. There are several key factors that companies may consider when evaluating and selecting green suppliers. These can include:

1. Environmental performance: This includes metrics such as greenhouse gas emissions, energy and water usage, and waste generation. Companies may look for suppliers that have demonstrated a commitment to improving their environmental performance over time.
2. Sustainability practices: Companies may evaluate the supplier’s sustainability practices, such as the use of renewable energy or resources, recycling and waste reduction efforts, and sustainable sourcing.
3. Compliance with environmental regulations: Companies may consider whether the supplier is compliant with relevant environmental regulations and standards.
4. Environmental policies and initiatives: Companies may look for suppliers that have developed and implemented environmental policies and initiatives, such as reducing their carbon footprint or adopting sustainable practices.
5. Reputation and track record: Companies may also consider the supplier’s reputation and track record when it comes to environmental performance and sustainability practices. By considering these factors, companies can identify suppliers that align with their own environmental and sustainability goals and help reduce the environmental impact of their supply chain.
6. There are several reasons why companies may choose to implement green supplier selection practices:
7. Cost savings: Adopting sustainable practices and sourcing from environmentally responsible suppliers can often result in cost savings through reduced energy and resource consumption, waste reduction, and improved efficiency.
8. Risk management: Choosing suppliers with strong environmental performance can help reduce the risk of environmental compliance issues and mitigate reputational risks.
9. Stakeholder expectations: Companies may choose to implement green supplier selection to meet the expectations of stakeholders such as customers, employees, and shareholders who are concerned about environmental and social issues.
10. Legal and regulatory requirements: In some cases, companies may be required by law or regulation to consider environmental performance in their supplier selection process.
11. Long-term competitiveness: Adopting sustainable practices and sourcing from green suppliers can help companies stay competitive in the long term, as consumers and other stakeholders increasingly demand environmentally responsible products and services.

### 1.3. Research gap

Despite the extensive research on green supply chain management (GSCM) and supplier selection, existing methods often rely on traditional fuzzy set theories, such as interval-valued Pythagorean fuzzy sets (IVPFS) and Pythagorean fuzzy sets (PFS), which may not fully capture the complex uncertainties inherent in real-world decision-making scenarios. While several studies have utilized these fuzzy set approaches to address multi-criteria decision-making (MCDM) problems, there remains a significant gap in the literature concerning the integration of cubic Pythagorean fuzzy sets (CPFS) in decision-making frameworks. CPFS offers a more comprehensive representation of uncertainty and imprecision by simultaneously addressing the limitations of IVPFS and PFS. However, there is a lack of research that leverages CPFS in conjunction with advanced decision-making techniques like the Technique for Order Preference by Similarity to Ideal Solution (TOPSIS) and nonlinear programming (NLP) models. This research addresses this gap by proposing a novel approach that integrates CPFS with TOPSIS and NLP models to improve the accuracy and robustness of green supplier selection (GSS) decisions. The study also contributes to the field by introducing interval weight information and developing algorithms that incorporate the relative closeness coefficient (RCC) and possibility degree, which have not been extensively explored in the context of CPFS.

### 1.4. Research questions

1. How can CPFS be effectively integrated into decision-making frameworks to improve the accuracy and reliability of green supplier selection (GSS) processes?
2. What are the advantages of combining the TOPSIS method with NLP models in a CPFS environment for solving complex decision-making problems (DMPs)?
3. How does the proposed CPFS-based approach compare with existing methods in terms of accuracy, efficiency, and ability to handle complex fuzzy decision-making problems?
4. What impact does the use of interval weight information and RCC have on the effectiveness of the proposed decision-making model?
5. How can the proposed algorithm be applied to real-world scenarios, such as green supplier selection, to validate its practical relevance and effectiveness?

### 1.5. Motivations

Driven by the unique structure of cubic Pythagorean fuzzy sets (CPFS) and the proven effectiveness of the TOPSIS method, this study introduces an innovative approach to solving decision-making problems (DMPs) by integrating TOPSIS with nonlinear programming (NLP) models. To the best of the authors’ knowledge, no prior research has explored DMPs under CPFS characteristics while incorporating interval weight information. This study breaks new ground by presenting a novel NLP approach, utilizing the TOPSIS method, to identify the most suitable green supplier within CPF environments. The primary goals of this research are to:

1. To represent information by incorporating the characteristics of CPFS: This objective aims to enhance the representation of uncertainty and imprecision in decision-making scenarios by integrating the complex features of CPFS. CPFS allows for more flexible modeling of membership and non-membership degrees compared to traditional fuzzy sets, leading to a more accurate and holistic depiction of real-world situations where ambiguity and vagueness are prevalent. By doing so, this approach captures the nuances of decision-making environments more effectively, particularly in cases where binary or crisp representations fall short.
2. To develop two NLP models using weighted distance measures: The second objective is to formulate robust NLP models that employ weighted distance measures to assess decision alternatives. These models offer a more comprehensive representation of the problem by accounting for both the relative importance of criteria and the inherent uncertainties in the data. Furthermore, the proposed NLP models are designed to be adaptable, including special cases that can be derived from them, making the approach versatile across different decision-making contexts. This flexibility enhances the model’s applicability to various problem types and complexities, ensuring broader usability in complex decision-making scenarios.
3. To introduce an algorithm that utilizes the relative closeness coefficient (RCC) and possibility degree to solve decision-making problems (DMPs): This objective focuses on developing an innovative algorithm that leverages RCC and possibility degree to rank and select the best decision alternatives. By incorporating RCC, the algorithm evaluates how close each alternative is to the ideal solution, providing a systematic approach for decision-making under uncertainty. Additionally, the use of possibility degree allows for the handling of fuzzy data more effectively, enhancing the algorithm’s capability to process and interpret ambiguous information. This method provides a structured and reliable approach for tackling complex DMPs, where precision is crucial.
4. To implement the proposed algorithm in a case study focused on GSS: The fourth objective involves applying the developed algorithm to a real-world case study related to green supplier selection. This application serves to validate the practicality and relevance of the approach in a critical area of supply chain management. By focusing on GSS, the study demonstrates how environmentally conscious decision-making can be integrated with advanced fuzzy modeling techniques. This case study illustrates the algorithm’s effectiveness in handling real-world challenges, such as balancing sustainability with other operational factors, providing insights into its potential impact on green supply chains.
5. To evaluate the performance of the proposed approach by comparing it with existing methods: The final objective is to rigorously assess the effectiveness of the proposed approach by benchmarking it against other established methods in the field. This evaluation includes a detailed comparison in terms of accuracy, efficiency, and the ability to handle complex, fuzzy decision-making problems. The goal is to demonstrate the superiority of the proposed method by highlighting its advantages, such as improved decision accuracy, better handling of uncertainty, and greater computational efficiency. This comparison not only validates the approach but also establishes its potential as a preferred method for solving intricate decision-making problems across various domains.

The remainder of the article is organized as follows: Section 2 provides an overview of fundamental theories including PFS, IVPFS, CS, CIFS, and CPFS. Section 3 presents a MCDM approach by developing the NLP model, incorporating the concept of RCC between pairs of CPFSs and their ideal values. In Section 4, we analyze the proposed models under various special cases. Section 5 demonstrates the application of the approach through a numerical example and includes a comparative analysis with existing studies. Finally, Section 6 presents the conclusions and implications of the findings.

## 2. Preliminaries

**Definition 1.** [34] For a non-empty fixed set *S*, the PFS is defined as

$$A=\{t,U_{A}(t),V_{A}(t)|t\in S\}$$ (1)

where $U_{A}:S\to [0,1]$ describes the MD and $V_{A}:S\to [0,1]$ describes the NMD of the component *t*∈*S* respectively, satisfying the condition $U_{A}^{2}(t)+U_{A}^{2}(t)≼1$ for every element *t*∈*S*. For the sake of simplicity $\left(U_{A}(t),V_{A}(t)\right)$ is referred to as a PFN, which can be represented as $\alpha =(U_{\alpha },V_{\alpha })$, satisfying the condition $U_{\alpha }^{2}+V_{\alpha }^{2}≼1$.

**Definition 2.** [35] Let *S* be a finite set, the IVPFS *B* over *S* is defined as

$$B=\{t,U_{B}(t),V_{B}(t)|t\in S\}$$ (2)

where $U_{B}(t)=[U_{B}^{-}(t),U_{B}^{+}(t)]\subseteq [0,1]$ and $V_{B}(t)=[V_{B}^{-}(t),V_{B}^{+}(t)]\subseteq [0,1]$ are interval values and $U_{B}^{-}(t)$, $U_{B}^{+}(t)$, $V_{B}^{-}(t)$, $V_{B}^{+}(t)\in [0,1]$, satisfying the condition ${\left(U_{B}^{+}(t)\right)}^{2}+{\left(V_{B}^{+}(t)\right)}^{2}≼1$. For the sake of simplicity, $\left(U_{B}(t),V_{B}(t)\right)$ is referred to as an IVPFN, which is represented by $\beta =(U_{\beta },V_{\beta })$, where $U_{\beta }=[U_{\beta }^{-},U_{\beta }^{+}]\subseteq [0,1]$ and $V_{\beta }=[V_{\beta }^{-},V_{\beta }^{+}]\subseteq [0,1]$, satisfying the condition ${\left(U_{\beta }^{+}\right)}^{2}+{\left(V_{\beta }^{+}\right)}^{2}≼1$. It should be mentioned that the IVPFN $\beta =(\left[U_{\beta }^{-},U_{\beta }^{+}],[V_{\beta }^{-},V_{\beta }^{+}\right])$ is referred to as the IVIFN if $U_{\beta }^{+}+V_{\beta }^{+}≼1$.

**Definition 3.** [36] Assume *S* is a finite set. In set *S*, CS can be defined as:

$$C=(t,\langle U_{C}(t),u_{C}(t)\rangle |t\in S)$$ (3)

where $U_{C}(t)=[U_{C}^{-},U_{C}^{+}]$ is an IVFS and $u_{C}(t)$ is a FS. For the sake of simplicity CS $\left(t,\langle U_{C}(t),u_{C}(t)\rangle \right)$ represented by $C=(U_{C},u_{C})$.

**Definition 4.** [36] Let *S* be a finite set. A CS $C=(U_{C},u_{C})$ is said to be an internal CS (ICS) if $U_{C}^{-}≼u_{C}≼U_{C}^{+}$ for all *t*∈*S*. On the other hand, a CS $C=(U_{C},u_{C})$ is said to be an external CS (ECS), if $u_{C}\in (U_{C}^{-},U_{C}^{+})\forall t\in S$.

**Definition 5.** [36] Let $C_{1}=(\xi_{C_{1}},\upsilon_{C_{1}})$ and $C_{2}=(\xi_{C_{2}},\upsilon_{C_{2}})$ be the two CSs, then

- (Equality) $C_{1}=C_{2}⟺U_{C_{1}}^{-}=U_{C_{2}}^{-},U_{C_{1}}^{+}=U_{C_{2}}^{+}$ and $u_{C_{1}}=u_{C_{2}}$.
- (P-order) $C_{1}\subseteq_{P}C_{2}⟺U_{C_{1}}^{-}≼U_{C_{2}}^{-},U_{C_{1}}^{+}≼U_{C_{2}}^{+}$ and $u_{C_{1}}≼u_{C_{2}}$.
- (R-order) $C_{1}\subseteq_{R}C_{2}⟺U_{C_{1}}^{-}≼U_{C_{2}}^{-},U_{C_{1}}^{+}≼U_{C_{2}}^{+}$ and $u_{C_{1}}≽u_{C_{1}}$.

**Definition 6.** [36] For any $C_{i}=\{t,\langle U_{C_{i}}(t),u_{C_{i}}(t)\rangle |t\in S\}$ where *i*∈Δ, then

- (P-union) $\overset{P}{\underset{i\in △}{⋃}}C_{i}=\{t,\langle \left(\underset{i\in △}{⋃}U_{C_{i}})(t),\;(\underset{i\in △}{⋁}u_{i})(t\right)\rangle |t\in S\}$
- (R-union) $\overset{R}{\underset{i\in △}{⋃}}C_{i}=\{t,\langle \left(\underset{i\in △}{⋃}U_{C_{i}})(t),\;(\underset{i\in △}{⋀}u_{i})(t\right)\rangle |t\in S\}$
- (P-intersection) ${⋂}_{i\in △}^{P}C_{i}=\{t,(\underset{i\in △}{⋂}U_{i})(t),\;(\underset{i\in △}{⋀}u_{i})(t)|t\in S\}$
- (R-intersection) ${⋂}_{i\in △}^{R}C_{i}=\{t,(\underset{i\in △}{⋂}U_{i})(t),\;(\underset{i\in △}{⋁}u_{i})(t)|t\in S\}$

**Definition 7.** [31,32] Let *S* be a non-empty set. A CIFS *F* constructed over the *S* is an ordered pair with the following description:

F={t,〈B(t),χ(t)〉|t∈S} (4)

where $B(t)=\{t,\langle \left[U_{B}^{-}(t),U_{B}^{+}(t)],[V_{B}^{-}(t),V_{B}^{+}(t)\right]\rangle |t\in S\}$ is IVIFS and while $\chi (t)=\{t,\langle U_{B}(t),V_{B}(t)\rangle |t\in S\}$ represents an IFS such that $0≼U_{B}^{-}(t)≼U_{B}^{+}(t)≼1$ and $0≼V_{B}^{-}(t)≼V_{B}^{+}(t)≼1$ satisfying the condition $U_{B}^{+}(t)+V_{B}^{+}(t)≼1$. Also $0≼U_{B}(t)≼V_{B}(t)≼1$ and satisfying the condition $U_{B}(t)+V_{B}(t)≼1$. For convenience, we will refer to these pairs as *F* = 〈*B*,χ〉, where $B=\langle \left[U_{B}^{-},U_{B}^{+}],[V_{B}^{-},V_{B}^{+}\right]\rangle$ and $\chi =\langle U_{B},V_{B})$ and also known as a CIF number (CIFN).

**Definition 8.** [31,32] Let *S* be a finite set. The CPFS over *S* is defined as

H={t,〈H(t),λ(t)〉|t∈S} (5)

where $H(t)=\langle t,[U_{H}^{-}(t),U_{H}^{+}(t)],[V_{H}^{-}(t),V_{H}^{+}(t)]|t\in S\rangle$ is IVPFS, while $\lambda (t)=\langle {t,U}_{H}(t),V_{H}(t)|t\in S\rangle$ represent PFS, such that $0≼U_{H}^{-}(t)≼U_{H}^{+}(t)≼1,0≼V_{H}^{-}(t)≼V_{H}^{+}(t)≼1$ and $0≼U_{H}(t),V_{H}(t)≼1$, satisfying the conditions ${\left(U_{H}^{+}(t)\right)}^{2}+{\left(V_{H}^{+}(t)\right)}^{2}≼1$ and ${\left(U_{H}(t)\right)}^{2}+{V_{H}(t)}^{2}≼1$. For convenience, we will refer to these pairs as ℋ = 〈*H*,*λ*〉, where $H=\langle \left[U_{H}^{-},U_{H}^{+}],[V_{H}^{-},V_{H}^{+}\right]\rangle$ and $\lambda =\langle U_{H},V_{H}\rangle$ and also known as a CPF number (CPFN).

**Definition 9.** [32] A CPFS ℋ = {*t*,〈*H*(*t*),*λ*(*t*)〉|*t*∈*S*} is said to be Internal CPFS if $U_{H}(t)\in [U_{H}^{-}(t),U_{H}^{+}(t)]$ and $V_{H}(t)=[V_{H}^{-}(t),V_{H}^{+}(t)]$ for all *t*∈*S*, otherwise called as external CPFS.

**Definition 10.** [20,33] For a family of CPFS {*F*_*i*_,*i*∈Δ}, we have

- ${⋃}_{i\in △}^{P}H_{i}=\left(\begin{array}{c} \langle \left[{max}_{i\in △}(U_{i}^{-}),{max}_{i\in △}(U_{i}^{+})],[{min}_{i\in △}(V_{i}^{-}),{min}_{i\in △}(V_{i}^{+})\right]\rangle ,\\ \langle {max}_{i\in △}(U_{i}),{min}_{i\in △}(V_{i})\rangle \end{array}\right)$,
- ${⋃}_{i\in △}^{R}H_{i}=\left(\begin{array}{c} \langle \left[{max}_{i\in △}(U_{i}^{-}),{max}_{i\in △}(U_{i}^{+})],[{min}_{i\in △}(V_{i}^{-}),{min}_{i\in △}(V_{i}^{+})\right]\rangle ,\\ \langle {min}_{i\in △}(U_{i}),{max}_{i\in △}(V_{i})\rangle \end{array}\right)$,
- ${⋂}_{i\in △}^{P}H_{i}=\left(\begin{array}{c} \langle \left[{min}_{i\in △}(U_{i}^{-}),{min}_{i\in △}(U_{i}^{+})],[{max}_{i\in △}(V_{i}^{-}),{max}_{i\in △}(V_{i}^{+})\right]\rangle ,\\ \langle {min}_{i\in △}(U_{i}),{max}_{i\in △}(V_{i})\rangle \end{array}\right)$,
- ${⋂}_{i\in △}^{R}H_{i}=\left(\begin{array}{c} \langle \left[{min}_{i\in △}(U_{i}^{-}),{min}_{i\in △}(U_{i}^{+})],[{max}_{i\in △}(V_{i}^{-}),{max}_{i\in △}(V_{i}^{+})\right]\rangle ,\\ \langle {max}_{i\in △}(U_{i}),{min}_{i\in △}(V_{i})\rangle \end{array}\right)$.

**Definition 11.** [20,33] For any two CPFNs $H_{i}=(\langle \left[U_{i}^{-},U_{i}^{+}],[V_{i}^{-},V_{i}^{+}\right]\rangle ,\langle U_{i},V_{i}\rangle )$ where (*i* = 1,2), we have

1. (Equality) $H_{1}=H_{2}⟺U_{1}^{-}=U_{2}^{-},U_{2}^{+}=U_{2}^{+},V_{1}^{-}=V_{2}^{-},V_{1}^{+}=V_{1}^{+},U_{1}=U_{2}$ and $V_{1}=V_{2}$
2. (P-order) ℋ_1_⊆_*P*_ ℋ_2_ if $[U_{1}^{-},U_{1}^{+}]\subseteq [U_{2}^{-},U_{2}^{+}],[V_{1}^{-},V_{1}^{+}]⊇[V_{2}^{-},V_{2}^{+}],U_{1}≼U_{2}$ and $V_{1}≽V_{2}$
3. (R-order) ℋ_1_⊆_*R*_ ℋ_2_ if $[U_{1}^{-},U_{1}^{+}]\subseteq [U_{2}^{-},U_{2}^{+}],[V_{1}^{-},V_{1}^{+}]⊇[V_{2}^{-},V_{2}^{+}],V_{1}≽V_{2}$ and $V_{1}≼V_{2}$

**Definition 12.** [37] Let $m=[m^{-},m^{+}]$, and $n=[n^{-},n^{+}]$ be any two interval numbers, then the likelihood of *δ*≽*τ* is defined as

$$p(m≽n)=max\left\{1-max\{\frac{n^{+}-m^{-}}{L(m)-L(n)},0),0\right\}$$ (6)

where $L(m)=m^{+}-m^{-}$ and $L(n)=n^{+}-n^{-}$.

## 3 Non-linear programming mechanism

This section explains how to solve DM problems using a TOPSIS technique based on the NLP simulations in an R-order CPFS context.

### 3.1 Description of the MCDM problem

The following is an example of typical MCDM problems in a CPFS context. Assume a collection of “*m*” different alternatives $s=[s_{1},s_{2},\ldots ,s_{m}]$ which can be evaluated using a set of “n” criteria *ζ* = {*ζ*_1_,*ζ*_2_,…,*ζ*_*n*_}. During the evaluation phase of each alternative, experts provide the rating about the alternative *ϑ*_*i*_ (*i* = 1,2,…,*m*) in terms of CPFS ℋ_*ij*_ = 〈*H*_*ij*_,*λ*_*ij*_〉, where $H_{ij}=\langle \left[U_{ij}^{-},U_{ij}^{+}],[V_{ij}^{-},V_{ij}^{+}\right]\rangle$ and $\lambda_{ij}=\langle U_{ij},V_{ij}\rangle$ reflects the score value of *i*^*th*^ alternative according to the criteria $r_{j}$. In this case, the portions $\left[U_{ij}^{-},U_{ij}^{+},\right]$ and $V_{ij}$ are the degree to which alternative $s_{i}$ agrees with the criterion $r_{j}$, whereas the portions $\left[\varphi_{ij}^{-},\varphi_{ij}^{+}\right]$ and *ξ*_*ij*_ specify alternative *ϑ*_*i*_′s disagreement with the criterion $r_{j}$. Thus, the aggregate choices for each alternative are put into the decision matrix and represented as $J={\left(H_{ij}\right)}_{m\times n}$.

Furthermore, it is observed that the varied criteria play an essential role during the assessments of the alternatives; thus, in real challenges, it is essential to consider their varying threshold values, rather than providing equal preferences. Suppose that an expert believes that the importance of MD while evaluating alternatives under the criterion $r_{j}$ varies from $w_{j}^{-}$ to $w_{j}^{+}$; on the other hand, the importance of NMD under the criterion $r_{j}$ varies from $v_{j}^{-}$ to $v_{j}^{+}$ such that $0≼w_{j}^{-}≼w_{j}^{+}≼1,0≼v_{j}^{-}≼v_{j}^{+}≼1$, satisfying the condition ${\left(w_{j}^{+}\right)}^{2}+{\left(v_{j}^{+}\right)}^{2}≼1$. As a result, the weight vector owned by the criteria $r_{j}$ corresponding to $s_{i}$ ratings are $\langle \left[w_{j}^{-},w_{j}^{+}],[v_{j}^{-},v_{j}^{+}\right]\rangle$. In the same way, the weight vector for the values *λ*_*ij*_ related with CPFNs can be sat as follows: $\langle \left[p_{ij}^{-},p_{ij}^{+}],[q_{ij}^{-},q_{ij}^{+}\right]\rangle$ where $0≼p_{ij}^{-},p_{ij}^{+},q_{ij}^{-},q_{ij}^{+}≼1$ such that ${\left(p_{ij}^{+}\right)}^{2}+{\left(q_{ij}^{+}\right)}^{2}≼1$. Hence, $W={\left(\left[w_{j}^{-},w_{j}^{+}],[v_{j}^{-},v_{j}^{+}\right]),(\left[p_{j}^{-},p_{j}^{+}],[q_{j}^{-},q_{j}^{+}\right]\right)}_{1\times n}$ may be used to indicate the total weight all of the criteria $r_{i}$.

### 3.2 Ideal solution quantity

The formation of ideal measurements also as reference points, is important in the DM process since it helps to choose the best alternative(s). Since the assessment values are captured in the format of CPFNs by decision matrix ℐ = (ℋ_*ij*_), the two ideals optimistic and pessimistic ideal solutions, represented by OIS and PIS respectively, may be considered as ℋ^+^ = (〈[1,1],[0,0]〉,〈0,1〉) and ℋ^−^ = (〈[0,0],[1,1]〉,〈1,0〉) in the CPFS context. It is obvious from these ideals that they are complementary to one another. Also, rather than restricting the MD and NMDs of ℋ^+^ and ℋ^−^ to the ideal conditions i.e., 1 or 0, an expert might alter them by defining these ideals as $H_{j}^{+}={\left(\langle \left[a_{j}^{+},a_{j}^{+}],[b_{j}^{+},b_{j}^{+}\right]\rangle ,\langle c_{j}^{+},d_{j}^{+}\rangle \right)}_{1\times n}$ and $H_{j}^{-}={\left(\langle \left[a_{j}^{-},a_{j}^{-}],[b_{j}^{-},b_{j}^{-}\right]\rangle ,\langle c_{j}^{-},d_{j}^{-}\rangle \right)}_{1\times n}$ where $a_{j}^{+}={max}_{j}\{U_{ij}^{+}\}b_{j}^{+}={min}_{j}\{V_{ij}^{+}\},c_{j}^{+}={min}_{j}\{U_{ij}\},d_{j}^{+}={max}_{j}\{V_{ij}\},a_{j}^{-}={min}_{j}\{U_{ij}^{-}\},b_{j}^{-}={max}_{j}\{V_{ij}^{+}\},c_{j}^{-}={max}_{j}\{U_{ij}\},d_{j}^{-}={min}_{j}\{V_{ij}\}$. As a result, it can be determined that $H_{j}^{-}≼(\langle \left[U_{ij}^{-},U_{ij}^{+}],[V_{ij}^{-},V_{ij}^{+}\right]\rangle ,\langle U_{ij},V_{ij}\rangle )≼H_{j}^{+}$.

### 3.3 Separation quantity values

To regulate quantity values between the alternative $s_{i}$ and its reference values ℋ^+^ and ℋ^−^, the weighted Euclidean measure for CPFNs is used [42]. For it, Suppose that for any $U_{ij}^{*}\in [U_{ij}^{-},U_{ij}^{+}],V_{ij}^{*}\in [V_{ij}^{-},V_{ij}^{+}],w_{ij}^{*}\in [w_{ij}^{-},w_{ij}^{+}],v_{ij}^{*}\in [v_{ij}^{-},v_{ij}^{+}],a_{ij}^{*}\in [a_{ij}^{-},a_{ij}^{+}]$ and $b_{ij}^{*}\in [b_{ij}^{-},b_{ij}^{+}]$. The separation measure of $s_{i}$ from ℋ^+^ = (〈[1,1],[0,0]〉,〈0,1〉) and ℋ^−^ = (〈[0,0],[1,1]〉,〈1,0〉) is defined as:

$$D(s_{i},H^{+})={\left(\sum_{j=1}^{n}\left[\begin{array}{c} \sqrt{w_{j}(1-{\left(U_{ij}^{*}\right)}^{2})}+v_{j}{\left(V_{ij}^{*}\right)}^{2}+v_{j}{\left(U_{ij}\right)}^{2}\\ +\sqrt{w_{j}(1-{\left(V_{ij}\right)}^{2})} \end{array}\right]\right)}^{\frac{1}{2}}$$ (7)

$$D(s_{i},H^{-})={\left(\sum_{j=1}^{n}\left[\begin{array}{c} w_{j}{\left(U_{ij}^{*}\right)}^{2}+\sqrt{v_{j}(1-{\left(V_{ij}^{*}\right)}^{2})}\\ +\sqrt{w_{j}(1-{\left(U_{ij}\right)}^{2})}+v_{j}{\left(V_{ij}\right)}^{2} \end{array}\right]\right)}^{\frac{1}{2}}$$ (8)

Likewise, if a decision maker makes use of $H_{j}^{+}={\left(\langle \left[a_{j}^{+},a_{j}^{+}],[b_{j}^{+},b_{j}^{+}\right]\rangle ,\langle c_{j}^{+},d_{j}^{+}\rangle \right)}_{1\times n}$ and $\rho_{j}^{-}={\left(\langle \left[a_{j}^{-},a_{j}^{-}],[b_{j}^{-},b_{j}^{-}\right]\rangle ,\langle c_{j}^{-},d_{j}^{1}\rangle \right)}_{1\times n}$, the quantity values expressed as:

$$D(s_{i},H^{+})={\left(\sum_{j=1}^{n}\left[\begin{array}{c} w_{j}{\left(p_{j}^{+}-U_{ij}^{*}\right)}^{2}+v_{j}{\left({q_{j}^{+}-V}_{ij}^{*}\right)}^{2}\\ +p_{j}{\left({a_{j}^{+}-U}_{ij}\right)}^{2}+q_{j}{\left(b_{j}^{+}-V_{ij}\right)}^{2} \end{array}\right]\right)}^{\frac{1}{2}}$$ (9)

$$D(s_{i},H^{-})={\left(\sum_{j=1}^{n}\left[\begin{array}{c} w_{j}{\left(p_{j}^{-}-U_{ij}^{*}\right)}^{2}+v_{j}{\left({q_{j}^{-}-V}_{ij}^{*}\right)}^{2}+\\ p_{j}{\left({a_{j}^{-}-U}_{ij}\right)}^{2}+q_{j}{\left(b_{j}^{-}-V_{ij}\right)}^{2} \end{array}\right]\right)}^{\frac{1}{2}}$$ (10)

### 3.4. Nature of the relative closeness coefficient

To assess the relative efficacy of the alternatives $s_{i}$ with respect to ℋ^+^ and ℋ^−^, we utilize Eq (11) to compute the closeness coefficients of the alternatives.

$$C_{i}({\left(U_{ij}^{*}\right)}_{m\times n},{\left(V_{ij}^{*}\right)}_{1\times n},{\left(w_{j}\right)}_{1\times n},{\left(v_{j}\right)}_{1\times n},{\left(p_{j}\right)}_{1\times n},{\left(q_{j}\right)}_{1\times n})$$

$$=\frac{D(s_{i},H^{+})}{D(s_{i},H^{-})+D(s_{i},H^{+})};\;d(s_{i},H^{+})\neq 0$$ (11)

where $U_{ij}^{*},V_{ij}^{*}$ are *m*×*n* matrices, and $w_{j},v_{j},p_{j}$ and $q_{j}$ are n-dimensional column weights for MD and NMD respectively. furthermore, $0≼(s_{i},H^{-})≼D(s_{i},H^{-})+D(s_{i},H^{+})$ and thus $0≼U_{ij}^{*},V_{ij}^{*},w_{j},v_{j},p_{j},q_{j}≼1$. *C_i_* is a function of 2*n*(*m*+2) unknown factors with continuous nature, which grows significantly even with small values of *m* and *n*, as shown in Eq (11). To clarify, if *m* = 3 and *n* = 4, i.e., two possibilities are assessed using Eq (11) to calculate the unknown factors, we have 40 unknown factors. If we examine a case with *m* = 5 and *n* = 6, however, the number of unknown factors grows to 84. As a result, the process becomes tedious, requiring significant effort, which increases computational time and adds to the costs. Hence, there is a critical need for a time-efficient technique to address this problem and reduce the number of unknown variables.

To accomplish this, we will first analyze the boundedness, continuity, and monotonicity of the function *C*_*i*_ with respect to the unknown factors $U_{ij}^{*}\in [U_{ij}^{-},U_{ij}^{+}],V_{ij}^{*}\in [V_{ij}^{-},V_{ij}^{+}]$. For this, we clarify the formulation of $C_{i}$ provided in Eq (11) using the measures provided in Eqs (7) and (8).


$$C_{i}=\frac{{\left(\sum_{j=1}^{n}\left[\sqrt{w_{j}(1-{\left(U_{ij}^{*}\right)}^{2})}+v_{j}{\left(V_{ij}^{*}\right)}^{2}+p_{j}{\left(U_{ij}\right)}^{2}+\sqrt{q_{j}(1-{\left(V_{ij}\right)}^{2})}\right]\right)}^{\frac{1}{2}}}{{\left(\sum_{j=1}^{n}\left[\begin{array}{c} \sqrt{w_{j}(1-{\left(U_{ij}^{*}\right)}^{2})}+v_{j}{\left(V_{ij}^{*}\right)}^{2}\\ +p_{j}{\left(\xi_{ij}\right)}^{2}+\sqrt{q_{j}(1-{\left(V_{ij}\right)}^{2})} \end{array}\right]\right)}^{\frac{1}{2}}+{\left(\sum_{j=1}^{n}\left[\begin{array}{c} \sqrt{w_{j}(1-{\left(U_{ij}^{*}\right)}^{2})}+v_{j}{\left(V_{ij}^{*}\right)}^{2}\\ +p_{j}{\left(U_{ij}\right)}^{2}+\sqrt{q_{j}(1-{\left(V_{ij}\right)}^{2})} \end{array}\right]\right)}^{\frac{1}{2}}}$$ (12)

To handle monotonic presentation, we partially differentiate about to $U_{ij}^{*}$ and obtain

$$\frac{\partial C_{i}}{\partial U_{ij}^{*}}=\frac{d(s_{i},\rho^{+})w_{j}(\xi_{ij}^{*})(1-D(s_{i},H^{-}))+D^{2}(\vartheta_{i},H^{-})w_{j}({1-U_{ij}^{*}}^{2})}{{\left(D^{-}(s_{i},H^{-})+D^{+}(s_{i},H^{+})\right)}^{2}.D(s_{i},H^{+})D(s_{i},H^{-})}$$ (13)

where $0≼D(s_{i},H^{+}),D(s_{i},H^{-})≼1$ and $0≼U_{ij}^{*}≼1$, which shows that $\frac{\partial C_{i}}{\partial U_{ij}^{*}}≻0$. However, for $w_{j}\neq 0,\frac{\partial C_{i}}{\partial U_{ij}^{*}}≻0$. *C_i_* is thus a monotonic and rising function of $U_{ij}^{*}$. Following the same procedure as for *C*_*i*_ to $V_{ij}^{*}$, the function is calculated to be:

$$\frac{\partial C_{i}}{\partial V_{ij}^{*}}=\frac{D(s_{i},\rho^{+})\omega_{j}(1-V_{ij}^{*})(1-D(s_{i},H^{-}))+D^{2}(s_{i},H^{-})\omega_{j}({V_{ij}^{*}}^{2})}{{\left(D^{-}(s_{i},H^{-})+D^{+}(s_{i},H^{+})\right)}^{2}.D(s_{i},H^{+})D(s_{i},H^{-})}$$ (14)

From $v_{j},p_{j},q_{j}\in [0,1]$, we follow that $\frac{\partial C_{i}}{\partial V_{ij}^{*}}≼1$, while if $v_{j}\neq 0,p_{j}\neq 0$ and $q_{j}\neq 0$, then $\frac{\partial C_{i}}{\partial V_{ij}^{*}}≼1$. As a result, *C*_*i*_ has a monotonic non-negative performance for $V_{ij}^{*}$. As *C*_*i*_ are continuous functions due to the closed and bounded character of the components $\xi_{ij}^{*},\xi_{ij},\varphi_{ij},w_{j},\omega_{j},\eta_{j}$ and *θ*_*j*_ of subinterval of [0,1]. Hence, the *C*_*i*_ values must likewise be the interval [0,1], denoted by $\left[C_{i}^{-},C_{i}^{+}\right]$. As a result of the fundamental concept as well as the function’s $C_{i}$ continuous nature, we have ${0≼C}_{i}^{-}≼C_{i}^{+}$ for $U_{ij}^{*}\in [U_{ij}^{-},U_{ij}^{+}]$ and so on. It may also be proven $C_{i}^{-}+(1-C_{i}^{+})=1-(C_{i}^{+}-C_{i}^{-})≼1$ and hence the set $\left[C_{i}^{-},C_{i}^{+}\right]$ may be written as a PFS $C_{i}=(C_{i}^{-},1-C_{i}^{+})$, indicating that the degree of closeness of alternatives *ϑ*_*i*_ to ℋ^+^ is $H_{i}^{-}$; whilst their non-closeness is $1-C_{i}^{+}$. Therefore, the ranks may be determined using the set $C_{i}=[C_{i}^{-},C_{i}^{+}]$.

### 3.5 Auxiliary NLP models

Since *C*_*i*_ has lower and upper bound because it is a limited and continuous function of $U_{ij}^{*},V_{ij}^{*},U_{ij},V_{ij}$. Therefore, by the continuity feature of *c*_*i*_, we may deduce that $C_{i}^{-}$ is obtained at the lower bounds of $V_{ij}^{*}$ and upper bound of $V_{ij}^{*}.C_{i}^{+}$, on the other hand, maybe initiate at both the lower and upper boundaries of $U_{ij}^{*}$. Thus, Eq (12) can be utilized to develop the two NLP models as follows:

$$C_{i}^{-}=min\left(\frac{{\left(\sum_{j=1}^{n}\left[\sqrt{w_{j}(1-{\left(U_{ij}^{*}\right)}^{2})}+v_{j}{\left(V_{ij}^{*}\right)}^{2}+q_{j}{\left(V_{ij}\right)}^{2}+\sqrt{q_{j}(1-{\left(V_{ij}\right)}^{2})}\right]\right)}^{\frac{1}{2}}}{{\left(\sum_{j=1}^{n}\left[\begin{array}{c} \sqrt{w_{j}(1-{\left(U_{ij}^{*}\right)}^{2})}+\omega_{j}{\left(V_{ij}^{*}\right)}^{2}\\ +p_{j}{\left(U_{ij}\right)}^{2}+\sqrt{q_{j}(1-{\left(V_{ij}\right)}^{2})} \end{array}\right]\right)}^{\frac{1}{2}}+{\left(\sum_{j=1}^{n}\left[\begin{array}{c} \sqrt{w_{j}(1-{\left(U_{ij}^{*}\right)}^{2})}+v_{j}{\left(V_{ij}^{*}\right)}^{2}\\ +p_{j}{\left(V_{ij}\right)}^{2}+\sqrt{q_{j}(1-{\left(V_{ij}\right)}^{2})} \end{array}\right]\right)}^{\frac{1}{2}}}\right)$$ (15)

and

$$C_{i}^{+}=max\left(\frac{{\left(\sum_{j=1}^{n}\left[\sqrt{w_{j}(1-{\left(U_{ij}^{*}\right)}^{2})}+v_{j}{\left(V_{ij}^{*}\right)}^{2}+p_{j}{\left(U_{ij}\right)}^{2}+\sqrt{q_{j}(1-{\left(V_{ij}\right)}^{2})}\right]\right)}^{\frac{1}{2}}}{{\left(\sum_{j=1}^{n}\left[\begin{array}{c} \sqrt{w_{j}(1-{\left(U_{ij}^{*}\right)}^{2})}+v_{j}{\left(V_{ij}^{*}\right)}^{2}\\ +p_{j}{\left(U_{ij}\right)}^{2}+\sqrt{q_{j}(1-{\left(V_{ij}\right)}^{2})} \end{array}\right]\right)}^{\frac{1}{2}}+{\left(\sum_{j=1}^{n}\left[\begin{array}{c} \sqrt{w_{j}(1-{\left(U_{ij}^{*}\right)}^{2})}+v_{j}{\left(V_{ij}^{*}\right)}^{2}\\ +p_{j}{\left(U_{ij}\right)}^{2}+\sqrt{q_{j}(1-{\left(V_{ij}\right)}^{2})} \end{array}\right]\right)}^{\frac{1}{2}}}\right)$$ (16)

Moreover, the RCC of alternatives $s_{i}$ can be obtained in $C_{i}=[C_{i}^{-},C_{i}^{+}]$, which leads to the inclusion-comparison probability of sets $C_{i}$ and $C_{k}$, represented by *p*(*C*_*i*_⊇*C*_*k*_) and thus, the likeliness of alternatives *p*(*C*_*i*_≽*C*_*k*_) = *p*(*C*_*i*_⊇*C*_*k*_), which is used to prioritize the alternatives, as described by Eq (6) as:

$$p(C_{i}≽C_{k})=p(C_{i}⊇C_{k})=max\left\{1-max\{\frac{C_{k}^{+}-C_{i}^{-}}{L(C_{i})+L(C_{k})},0\},0\right\}$$ (17)

where $L(C_{i})=C_{k}^{+}-C_{i}^{-}$ and $L(C_{k})=C_{k}^{+}-C_{k}^{-}$. Therefore, the likelihood matrix is denoted by $P={\left(p^{ik}\right)}_{m\times n}$ where $p^{ik}=p(s_{i}≽s_{k})$. Also, interesting is the conclusion that 0≼*p*^*ik*^≼1 and *p*^*ik*^+*p*^*ki*^ = 1. The ideal value of each alternative is designed as

$$D_{i}=\frac{1}{m(m-1)}(\sum_{k=1}^{m}p^{ik}+\frac{m}{2}-1)$$ (18)

Next, the alternatives are ranked based on the decreasing values of $D_{i}$, and the best alternative(s) is selected accordingly.

The various phases involved in the TOPSIS technique, which relies on the NLP approach, are outlined in Algorithm 1 as described in the study above.

### 3.6. Algorithm

1. Describe the assessed material for each alternative $s_{i}$ provided by the experts in the CPFS context as $H=(\langle \left[U_{ij}^{-},U_{ij}^{+}],[V_{ij}^{-},V_{ij}^{+}\right]\rangle ,\langle U_{ij},V_{ij}\rangle )$.
2. Establish the importance of each criterion $r_{j}$ in the light of their relative weights as $W={\left(\left[w_{j}^{-},w_{j}^{+}],[v_{j}^{-},v_{j}^{+}\right]),(\left[p_{j}^{-},p_{j}^{+}],[q_{j}^{-},q_{j}^{+}\right]\right)}_{1\times n}$.
3. Calculate the optimization representations (15) and (16) for each alternative $s_{i}$ and fix their RCCs in terms of interval sets $C_{i}=[C_{i}^{-},C_{i}^{+}]$.
4. Construct a likelihood matrix by using Eq (17) as follows:
$$A=\left(\begin{array}{ccc} p^{11} & \cdots & p^{1m}\\ \vdots & \ddots & \vdots \\ p^{m1} & \cdots & p^{mm} \end{array}\right)$$
  where $p^{ik}=p(s_{i}≽s_{k})$, and hence Eq (18) is used to calculate the values of $D_{i}$.
5. Select the most ideal alternative based on the decreasing value of $D_{i}$.

## 4. Other properties of the presented NPL models

Eqs (15) and (16) possess additional properties, which are outlined below:

1. It is important to recognize that the evaluation in Section 3.4 relies on fixed ℋ^+^ and ℋ^−^ values, which means that the relative closeness coefficients (RCCs) remain unchanged regardless of the alternative considered. To fully address this issue, we have updated the ideal values to $H_{j}^{+}={\left(\langle \left[a_{j}^{+},a_{j}^{+}],[b_{j}^{+},b_{j}^{+}\right]\rangle ,\langle c_{j}^{+},d_{j}^{+}\rangle \right)}_{1\times n}$ and $H_{j}^{-}={\left(\langle \left[a_{j}^{-},a_{j}^{-}],[b_{j}^{-},b_{j}^{-}\right]\rangle ,\langle c_{j}^{-},d_{j}^{-}\rangle \right)}_{1\times n}$ in the formulas provided in Eqs (9) and (10). The RCC for the alternatives $s_{i}$ is then determined using these revised ideal values.
$$C_{i}=\frac{{\left(\sum_{j=1}^{n}\left[w_{j}{\left(a_{j}^{-}-U_{ij}^{*}\right)}^{2}+v_{j}{\left({b_{j}^{-}-V}_{ij}^{*}\right)}^{2}+p_{j}{\left({c_{j}^{-}-U}_{ij}\right)}^{2}+q_{j}{\left(d_{j}^{-}-V_{ij}\right)}^{2}\right]\right)}^{\frac{1}{2}}}{{\left(\sum_{j=1}^{n}\left[\begin{array}{c} w_{j}{\left(a_{j}^{-}-U_{ij}^{*}\right)}^{2}+\omega_{j}{\left({s_{j}^{-}-V}_{ij}^{*}\right)}^{2}+\\ p_{j}{\left({c_{j}^{-}-U}_{ij}\right)}^{2}+q_{j}{\left(d_{j}^{-}-V_{ij}\right)}^{2} \end{array}\right]\right)}^{\frac{1}{2}}+{\left(\sum_{j=1}^{n}\left[\begin{array}{c} w_{j}{\left(a_{j}^{+}-U_{ij}^{*}\right)}^{2}+\omega_{j}{\left({b_{j}^{+}-V}_{ij}^{*}\right)}^{2}+\\ p_{j}{\left({c_{j}^{+}-U}_{ij}\right)}^{2}+q_{j}{\left(d_{j}^{+}-V_{ij}\right)}^{2} \end{array}\right]\right)}^{\frac{1}{2}}}$$ (19)
  and hence, by models (15) and (16), $C_{i}^{-}$ and $C_{i}^{+}$ can be calculated as:
$$C_{i}^{-}=C_{i}({\left(U_{ij}^{-}\right)}_{m\times n},{\left(V_{ij}^{-}\right)}_{1\times n},{\left(w_{j}\right)}_{1\times n},{\left(v_{j}\right)}_{1\times n},{\left(p_{j}\right)}_{1\times n},{\left(q_{j}\right)}_{1\times n})$$ (20)
  and
$$C_{i}^{+}=C_{i}({\left(U_{ij}^{+}\right)}_{m\times n},{\left(V_{ij}^{+}\right)}_{1\times n},{\left(w_{j}\right)}_{1\times n},{\left(v_{j}\right)}_{1\times n},{\left(p_{j}\right)}_{1\times n},{\left(q_{j}\right)}_{1\times n})$$ (21)
  where $w_{j}^{-}≼w_{j}≼w_{j}^{+},v_{j}^{-}≼v_{j}≼v_{j}^{+},p_{j}^{-}≼p_{j}≼p_{j}^{+}$, and $q_{j}^{-}≼q_{j}≼q_{j}^{+}$ for all *j* = 1,2,…,*n*.
2. When we apply the weighted Hamming distance to calculate the distance between the alternative and the ideal solutions, we obtain:
$$D(s_{i},H^{+})=\sum_{j=1}^{n}\left\{\begin{array}{c} \sqrt{w_{j}(1-{\left(U_{ij}^{*}\right)}^{2})}+v_{j}{\left(V_{ij}^{*}\right)}^{2}\\ +p_{j}{\left(U_{ij}\right)}^{2}+\sqrt{p_{j}(1-{\left(V_{ij}\right)}^{2})} \end{array}\right\}$$ (22)
$$D(s_{i},H^{-})=\sum_{j=1}^{n}\left\{\begin{array}{c} w_{j}U_{ij}^{*}+\sqrt{v_{j}(1-{\left(V_{ij}^{*}\right)}^{2})}\\ +\sqrt{p_{j}(1-{\left(U_{ij}\right)}^{2})}+q_{j}V_{ij} \end{array}\right\}$$ (23)
  Hence, the RCC of $s_{i}$ can be expressed as
$$C_{i}=\frac{\sum_{j=1}^{n}\left\{w_{j}U_{ij}^{*}+\sqrt{v_{j}(1-{\left(V_{ij}^{*}\right)}^{2})}+\sqrt{p_{j}(1-{\left(U_{ij}\right)}^{2})}+q_{j}V_{ij}\right\}}{\sum_{j=1}^{n}\left(w_{j}+v_{j}+p_{j}+q_{j}\right)}$$ (24)
  and,
$$C_{i}^{-}=min\left(\frac{\sum_{j=1}^{n}\left\{w_{j}U_{ij}^{-}+\sqrt{v_{j}(1-{\left(V_{ij}^{+}\right)}^{2})}+\sqrt{p_{j}(1-{\left(V_{ij}\right)}^{2})}+q_{j}V_{ij}\right\}}{\sum_{j=1}^{n}\left(w_{j}+v_{j}+p_{j}+q_{j}\right)}\right)$$ (25)
$$C_{i}^{+}=max\left(\frac{\sum_{j=1}^{n}\left\{w_{j}U_{ij}^{+}+\sqrt{v_{j}(1-{\left(V_{ij}^{-}\right)}^{2})}+\sqrt{p_{j}(1-{\left(U_{ij}\right)}^{2})}+q_{j}V_{ij}\right\}}{\sum_{j=1}^{n}\left(w_{j}+v_{j}+p_{j}+q_{j}\right)}\right)$$ (26)
3. The suggested model is applicable to various MCDM problems where the criterion weights are known in advance and given as a positive constant $w_{j}≻0$. The range of the RCC can be identified as follows:
$$C_{i}^{-}=\frac{1}{4}\sum_{j=1}^{n}w_{j}\{U_{ij}^{-}+\sqrt{1-V_{ij}^{+}}+\sqrt{1-U_{ij}}+V_{ij}\}$$ (27)
  and
$$C_{i}^{+}=\frac{1}{4}\sum_{j=1}^{n}w_{j}\{U_{ij}^{+}+\sqrt{1-V_{ij}^{-}}+\sqrt{1-U_{ij}}+V_{ij}\}$$ (28)
  respectively.
4. If $H_{j}^{+}=(\langle \left[a_{j}^{+},a_{j}^{+}],[b_{j}^{+},b_{j}^{+}\right]\rangle ,\langle c_{j}^{+},d_{j}^{+}\rangle )$ and $H_{j}^{-}=(\langle \left[a_{j}^{-},a_{j}^{-}],[b_{j}^{-},b_{j}^{-}\right]\rangle ,\langle c_{j}^{-},d_{j}^{-}\rangle )$ then the bounds of RCCs can be obtained as
$$C_{i}^{-}=\frac{\sum_{j=1}^{n}\left(w_{j}(U_{ij}^{-}-a_{j}^{-})+v_{j}(b_{j}^{-}-V_{ij}^{+})+p_{j}(c_{j}^{-}-U_{ij})+q_{j}(V_{ij}-d_{j}^{-})\right)}{\sum_{j=1}^{n}\left(w_{j}(a_{j}^{+}-a_{j}^{-})+v_{j}(b_{j}^{-}-b_{j}^{+})+p_{j}(c_{j}^{-}-c_{j}^{+})+q_{j}(d_{j}^{+}-d_{j}^{-})\right)}$$ (29)
$$C_{i}^{+}=\frac{\sum_{j=1}^{n}\left(w_{j}(U_{ij}^{+}-a_{j}^{-})+v_{j}(b_{j}^{-}-V_{ij}^{-})+p_{j}(c_{j}^{-}-U_{ij})+q_{j}(V_{ij}-d_{j}^{-})\right)}{\sum_{j=1}^{n}\left(w_{j}(a_{j}^{+}-a_{j}^{-})+v_{j}(b_{j}^{-}-b_{j}^{+})+p_{j}(c_{j}^{-}-c_{j}^{+})+q_{j}(d_{j}^{+}-d_{j}^{-})\right)}$$ (30)
  respectively.

## 5. Application

Supply chain strategy is the foundation of green supply chain management, which is a major element of the environmentally friendly supply chain. The efficiency of the entire supply network is directly influenced by the ability of the suppliers. The company needs to find a rapid and efficient solution to the issue of choosing the appropriate suppliers. Numerous academics contend that when picking suppliers, it is important to consider both the product quality and service standards of each provider, whether they are conventional or green. The processing and manufacturing of enterprises depend on the quality of items offered by the supplier, which affects the market efficiency of the products of an enterprise and dictates the efficiency of the manufacturing and quality of the concluding product. The effectiveness of the supplier in solving issues and delivering products is reflected in the service level, which is a crucial benchmark for the enterprise to use when determining whether the supply of raw materials can be successfully assured. Additionally, businesses must consider the environmental protection approach, quality of green energy, and other factors when choosing green suppliers. The identification of green suppliers is a well-known MAGDM issue [38,39]. Based on this fundamental investigation and survey regarding green supplier selection, assume that the management chooses the four characteristics listed below to review and pick the appropriate green supplier(s): (1) $Y_{1}$ is the team’s skill; (2) $Y_{2}$ is the green degree of design and production; and (3) $Y_{3}$ is the expenditure of waste disposal. The five experts ($E_{1},E_{2},E_{3},E_{4}$, and $E_{5}$) individually assessed the four green suppliers ($D_{1},D_{2},D_{3}$, and $D_{4}$) based on the three characteristics listed above and constructed the following five cubic Pythagorean fuzzy (CPF) decision matrices which are shown in Tables 1–5.

**Table 1:** Assessments of decision maker $E_{1}$.

$D_{i}$	$Y_{1}$	$Y_{2}$	$Y_{3}$
$D_{1}$	$\left(\begin{array}{c} \langle \left[0.4,0.5],[0.3,0.4\right]\rangle ,\\ \left(0.5,0.4\right) \end{array}\right)$	$\left(\begin{array}{c} \langle \left[0.4,0.6],[0.2,0.5\right]\rangle ,\\ \left(0.3,0.4\right) \end{array}\right)$	$\left(\begin{array}{c} \langle \left[0.3,0.5],[0.4,0.5\right]\rangle ,\\ \left(0.1,0.6\right) \end{array}\right)$
$D_{2}$	$\left(\begin{array}{c} \langle \left[0.3,0.4],[0.2,0.3\right]\rangle ,\\ \left(0.2,0.5\right) \end{array}\right)$	$\left(\begin{array}{c} \langle \left[0.1,0.3],[0.6,0.7\right]\rangle ,\\ \left(0.4,0.2\right) \end{array}\right)$	$\left(\begin{array}{c} \langle \left[0.4,0.7],[0.3,0.4\right]\rangle ,\\ \left(0.3,0.6\right) \end{array}\right)$
$D_{3}$	$\left(\begin{array}{c} \langle \left[0.6,0.7],[0.1,0.2\right]\rangle ,\\ \left(0.6,0.4\right) \end{array}\right)$	$\left(\begin{array}{c} \langle \left[0.2,0.4],[0.5,0.7\right]\rangle ,\\ \left(0.2,0.7\right) \end{array}\right)$	$\left(\begin{array}{c} \langle \left[0.3,0.4],[0.4,0.7\right]\rangle ,\\ \left(0.1,0.4\right) \end{array}\right)$
$D_{4}$	$\left(\begin{array}{c} \langle \left[0.3,0.5],[0.4,0.5\right]\rangle ,\\ \left(0.1,0.3\right) \end{array}\right)$	$\left(\begin{array}{c} \langle \left[0.6,0.8],[0.1,0.3\right]\rangle ,\\ \left(0.2,0.8\right) \end{array}\right)$	$\left(\begin{array}{c} \langle \left[0.1,0.4],[0.5,0.6\right]\rangle ,\\ \left(0.4,0.6\right) \end{array}\right)$

**Table 2:** Assessments of decision maker $E_{2}$.

$D_{i}$	$Y_{1}$	$Y_{2}$	$Y_{3}$
$D_{1}$	$\left(\begin{array}{c} \langle \left[0.2,0.4],[0.5,0.6\right]\rangle ,\\ \left(0.3,0.5\right) \end{array}\right)$	$\left(\begin{array}{c} \langle \left[0.3,0.4],[0.2,0.3\right]\rangle ,\\ \left(0.5,0.8\right) \end{array}\right)$	$\left(\begin{array}{c} \langle \left[0.1,0.4],[0.4,0.5\right]\rangle ,\\ \left(0.4,0.3\right) \end{array}\right)$
$D_{2}$	$\left(\begin{array}{c} \langle \left[0.4,0.6],[0.6,0.7\right]\rangle ,\\ \left(0.4,0.6\right) \end{array}\right)$	$\left(\begin{array}{c} \langle \left[0.4,0.5],[0.6,0.8\right]\rangle ,\\ \left(0.1,0.2\right) \end{array}\right)$	$\left(\begin{array}{c} \langle \left[0.3,0.6],[0.2,0.3\right]\rangle ,\\ \left(0.6,0.4\right) \end{array}\right)$
$D_{3}$	$\left(\begin{array}{c} \langle \left[0.6,0.7],[0.1,0.2\right]\rangle ,\\ \left(0.5,0.7\right) \end{array}\right)$	$\left(\begin{array}{c} \langle \left[0.3,0.4],[0.1,0.2\right]\rangle ,\\ \left(0.6,0.4\right) \end{array}\right)$	$\left(\begin{array}{c} \langle \left[0.3,0.4],[0.5,0.6\right]\rangle ,\\ \left(0.3,0.7\right) \end{array}\right)$
$D_{4}$	$\left(\begin{array}{c} \langle \left[0.3,0.6],[0.4,0.5\right]\rangle ,\\ \left(0.3,0.5\right) \end{array}\right)$	$\left(\begin{array}{c} \langle \left[0.6,0.7],[0.3,0.4\right]\rangle ,\\ \left(0.8,0.1\right) \end{array}\right)$	$\left(\begin{array}{c} \langle \left[0.4,0.5],[0.3,0.4\right]\rangle ,\\ \left(0.4,0.3\right) \end{array}\right)$

**Table 3:** Assessments of decision maker $E_{3}$.

$D_{3}$	$Y_{1}$	$Y_{2}$	$Y_{3}$
$D_{1}$	$\left(\begin{array}{c} \langle \left[0.1,0.2],[0.4,0.6\right]\rangle ,\\ \left(0.2,0.7\right) \end{array}\right)$	$\left(\begin{array}{c} \langle \left[0.4,0.5],[0.6,0.7\right]\rangle ,\\ \left(0.6,0.3\right) \end{array}\right)$	$\left(\begin{array}{c} \langle \left[0.3,0.4],[0.1,0.3\right]\rangle ,\\ \left(0.4,0.5\right) \end{array}\right)$
$D_{2}$	$\left(\begin{array}{c} \langle \left[0.3,0.4],[0.2,0.3\right]\rangle ,\\ \left(0.1,0.8\right) \end{array}\right)$	$\left(\begin{array}{c} \langle \left[0.1,0.4],[0.2,0.3\right]\rangle ,\\ \left(0.4,0.1\right) \end{array}\right)$	$\left(\begin{array}{c} \langle \left[0.5,0.6],[0.2,0.4\right]\rangle ,\\ \left(0.3,0.6\right) \end{array}\right)$
$D_{3}$	$\left(\begin{array}{c} \langle \left[0.5,0.6],[0.4,0.5\right]\rangle ,\\ \left(0.2,0.1\right) \end{array}\right)$	$\left(\begin{array}{c} \langle \left[0.2,0.5],[0.6,0.7\right]\rangle ,\\ \left(0.3,0.4\right) \end{array}\right)$	$\left(\begin{array}{c} \langle \left[0.4,0.5],[0.3,0.4\right]\rangle ,\\ \left(0.2,0.8\right) \end{array}\right)$
$D_{4}$	$\left(\begin{array}{c} \langle \left[0.3,0.4],[0.7,0.8\right]\rangle ,\\ \left(0.5,0.4\right) \end{array}\right)$	$\left(\begin{array}{c} \langle \left[0.5,0.6],[0.2,0.5\right]\rangle ,\\ \left(0.5,0.7\right) \end{array}\right)$	$\left(\begin{array}{c} \langle \left[0.1,0.2],[0.5,0.7\right]\rangle ,\\ \left(0.4,0.5\right) \end{array}\right)$

**Table 4:** Assessments of decision maker $E_{4}$.

$D_{i}$	$Y_{1}$	$Y_{2}$	$Y_{3}$
$D_{1}$	$\left(\begin{array}{c} \langle \left[0.5,0.6],[0.2,0.3\right]\rangle ,\\ \left(0.4,0.5\right) \end{array}\right)$	$\left(\begin{array}{c} \langle \left[0.1,0.2],[0.6,0.7\right]\rangle ,\\ \left(0.4,0.2\right) \end{array}\right)$	$\left(\begin{array}{c} \langle \left[0.6,0.7],[0.3,0.4\right]\rangle ,\\ \left(0.4,0.2\right) \end{array}\right)$
$D_{2}$	$\left(\begin{array}{c} \langle \left[0.4,0.5],[0.2,0.4\right]\rangle ,\\ \left(0.3,0.5\right) \end{array}\right)$	$\left(\begin{array}{c} \langle \left[0.4,0.5],[0.3,0.4\right]\rangle ,\\ \left(0.6,0.2\right) \end{array}\right)$	$\left(\begin{array}{c} \langle \left[0.3,0.4],[0.1,0.3\right]\rangle ,\\ \left(0.6,0.1\right) \end{array}\right)$
$D_{3}$	$\left(\begin{array}{c} \langle \left[0.6,0.7],[0.1,0.3\right]\rangle ,\\ \left(0.7,0.3\right) \end{array}\right)$	$\left(\begin{array}{c} \langle \left[0.2,0.4],[0.4,0.6\right]\rangle ,\\ \left(0.3,0.5\right) \end{array}\right)$	$\left(\begin{array}{c} \langle \left[0.4,0.5],[0.2,0.3\right]\rangle ,\\ \left(0.3,0.4\right) \end{array}\right)$
$D_{4}$	$\left(\begin{array}{c} \langle \left[0.3,0.4],[0.5,0.6\right]\rangle ,\\ \left(0.3,0.2\right) \end{array}\right)$	$\left(\begin{array}{c} \langle \left[0.2,0.5],[0.3,0.4\right]\rangle ,\\ \left(0.1,0.8\right) \end{array}\right)$	$\left(\begin{array}{c} \langle \left[0.2,0.3],[0.5,0.6\right]\rangle ,\\ \left(0.7,0.4\right) \end{array}\right)$

**Table 5:** Assessments of decision maker $E_{5}$.

$D_{i}$	$Y_{1}$	$Y_{2}$	$Y_{3}$
$D_{1}$	$\left(\begin{array}{c} \langle \left[0.7,0.8],[0.2,0.3\right]\rangle ,\\ \left(0.4,0.6\right) \end{array}\right)$	$\left(\begin{array}{c} \langle \left[0.2,0.3],[0.4,0.5\right]\rangle ,\\ \left(0.5,0.4\right) \end{array}\right)$	$\left(\begin{array}{c} \langle \left[0.2,0.5],[0.3,0.5\right]\rangle ,\\ \left(0.3,0.7\right) \end{array}\right)$
$D_{2}$	$\left(\begin{array}{c} \langle \left[0.4,0.6],[0.2,0.3\right]\rangle ,\\ \left(0.1,0.2\right) \end{array}\right)$	$\left(\begin{array}{c} \langle \left[0.2,0.6],[0.1,0.3\right]\rangle ,\\ \left(0.1,0.5\right) \end{array}\right)$	$\left(\begin{array}{c} \langle \left[0.1,0.3],[0.3,0.4\right]\rangle ,\\ \left(0.2,0.3\right) \end{array}\right)$
$D_{3}$	$\left(\begin{array}{c} \langle \left[0.1,0.3],[0.3,0.4\right]\rangle ,\\ \left(0.6,0.4\right) \end{array}\right)$	$\left(\begin{array}{c} \langle \left[0.5,0.6],[0.2,0.3\right]\rangle ,\\ \left(0.2,0.7\right) \end{array}\right)$	$\left(\begin{array}{c} \langle \left[0.3,0.4],[0.2,0.3\right]\rangle ,\\ \left(0.4,0.6\right) \end{array}\right)$
$D_{4}$	$\left(\begin{array}{c} \langle \left[0.3,0.5],[0.2,0.4\right]\rangle ,\\ \left(0.2,0.6\right) \end{array}\right)$	$\left(\begin{array}{c} \langle \left[0.1,0.2],[0.3,0.5\right]\rangle ,\\ \left(0.6,0.5\right) \end{array}\right)$	$\left(\begin{array}{c} \langle \left[0.3,0.4],[0.4,0.5\right]\rangle ,\\ \left(0.6,0.4\right) \end{array}\right)$

Based on this information, the proposed algorithm that utilizes CPFNs to select the optimal alternative(s) is advantageous. Algorithm 1’s steps are executed in the following phases:

**Phase 2.** The complete information about each criterion is summarized in Table 6.

**Table 6:** Subjective weights of each criterion.

$G_{j}$	$W_{j}$
$G_{1}$	(([0.1,0.4],[0.2,0.55]),([0.1,0.3],[0.2,0.6]))
$G_{2}$	(([0.2,0.5],[0.15,0.45]),([0.2,0.5],[0.3,0.5]))
$G_{3}$	(([0.2,0.5],[0.15,0.38]),([0.1,0.4],[0.2,0.3]))

**Phase 3.** To solve Eqs (22–24) we have $C_{1}^{-}=0.2114\;\mathrm{and}\;C_{1}^{+}=0.4111$, and thus, the RCC for alternative $D_{1}$ is *C*_1_ = [0.2114.0.4111]. Likewise, by applying these Equations, the comparative values of Remaining alternatives are obtained as *C*_2_ = [0.3516,0.5678], *C*_3_ = [0,2651,0.4637] and *C*_4_ = [0.3112,0.5483].

**Phase 4.** Applying Eqs (17) and (18), the possible matrix of options is formed as shown below:

$$p=\begin{array}{c} D_{1}\\ D_{2}\\ D_{3}\\ D_{4} \end{array}(\begin{array}{cccc} 0.5000 & 0.0979 & 0.2676 & 0.1936\\ 0.9021 & 0.5000 & 0.7155 & 0.4955\\ 0.7324 & 0.2845 & 0.5000 & 0.3248\\ 0.8064 & 0.5043 & 0.6742 & 0.5000 \end{array}).$$

**Fig 1:**
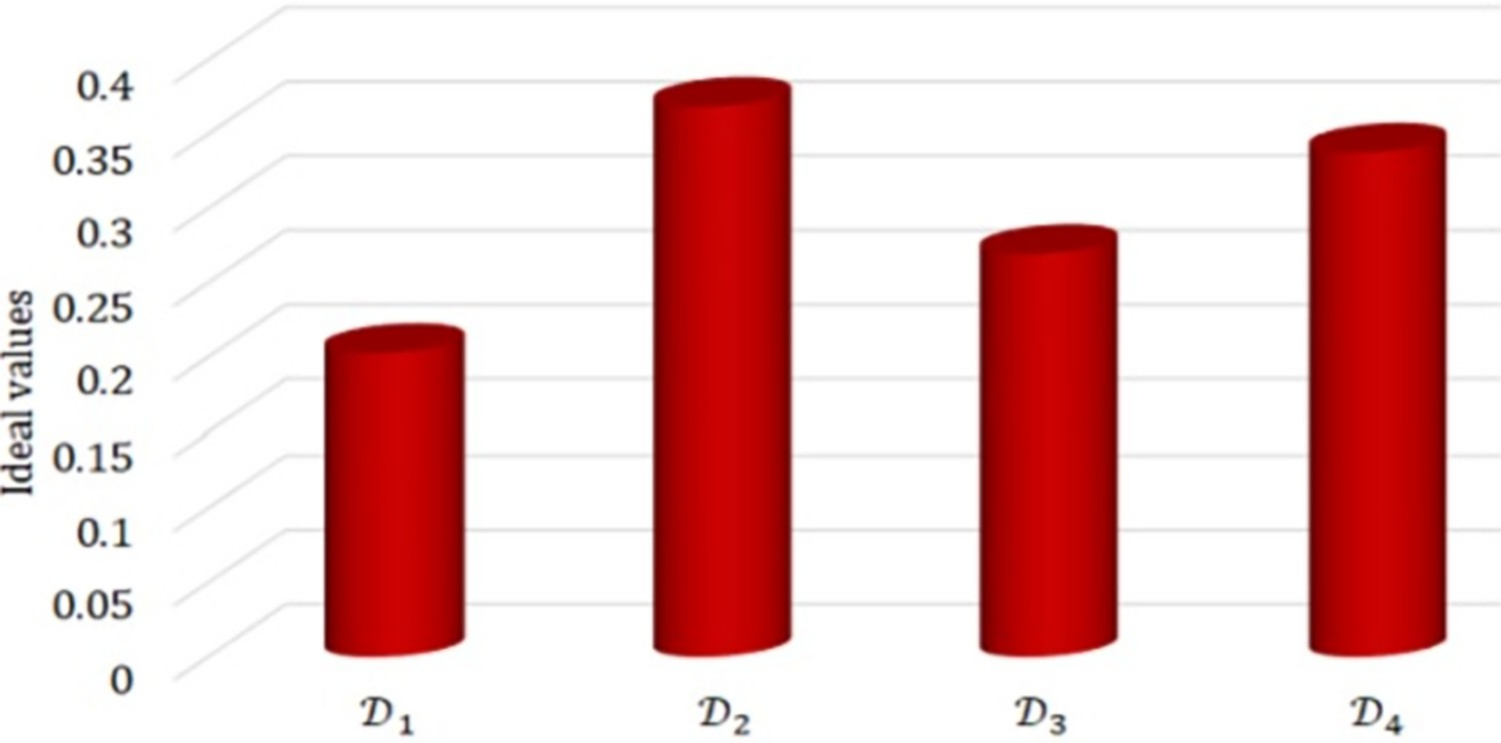
The graphical view of ideal values.

By Eq (18), we have $D_{1}=0.2027,D_{2}=0.3684,D_{3}=0.2694$ and $D_{4}=0.3376$. Fig 1 presents a graphical representation of the ideal values.

**Phase 5.** The ranking of alternatives is $D_{2}≻D_{4}≻D_{3}≻D_{1}$, and hence $D_{2}$ is the optimal alternative.

By treating each criterion’s weight vector as a known real number rather than an interval. Then, to execute the steps of Algorithm 1, we returned to the previous example, and the steps are summarized as follows:

**Step 1.** The evaluation values possessed by alternatives are formulated in Tables 1–5.

**Step 2.** Suppose that the weight vector of the criterion is *w* = (0.30,0.50,0.20)^*T*^.

**Step 3.** By Eqs (25) and (26), the RCCs interval of the alternatives are *C*_1_ = [0.3734,0.451], *C*_2_ = [0.5530,0.6065], *C*_3_ = [0.5037,0.5512] and *C*_4_ = [0.5378,0.6314].

Utilizing Eq (25) to determine the likelihood probability values, which are computed as

$$p=\begin{array}{c} D_{1}\\ D_{2}\\ D_{3}\\ D_{4} \end{array}(\begin{array}{c} 0.5000\;0.0000\;0.0000\;0.0000\\ 1.0000\;0.5000\;1.0000\;0.4970\\ 1.0000\;0.0000\;0.5000\;0.1264\\ 1.0000\;0.5030\;0.8734\;0.5000 \end{array})$$

By Eq (25) we get $D_{1}=0.1841,D_{2}=0.4183,D_{3}=0.2934$ and $D_{4}=0.3217$.

**Fig 2:**
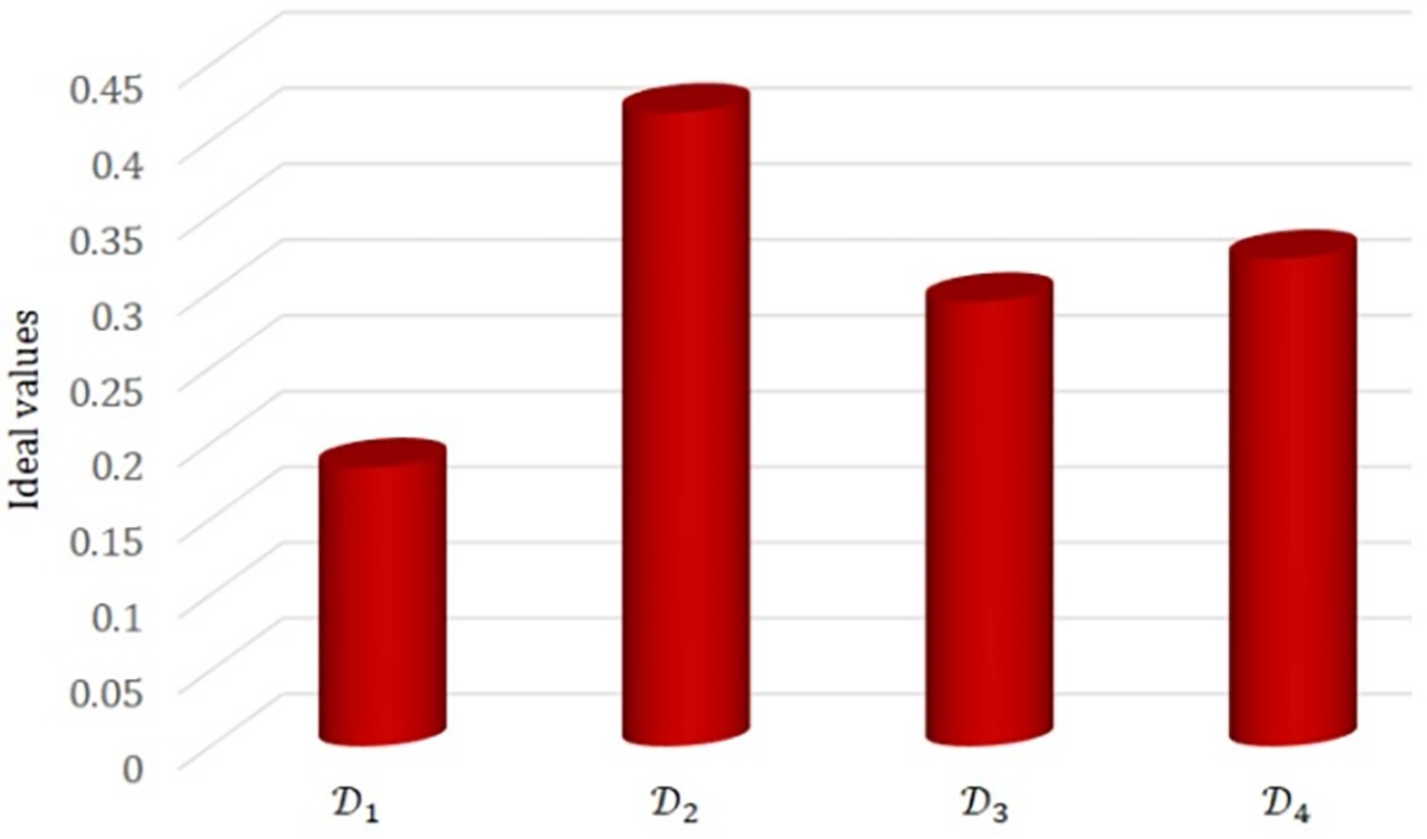
The ideal values of alternatives with weight vector *w* = (0.30,0.50,0.20)^*T*^.

Fig 2 displays a graphical representation of the ideal values.

**Step 5.** The position order of alternatives is $D_{2}≻D_{4}≻D_{3}≻D_{1}$, and hence $D_{2}$ is the optimal alternative.

## 6. Comparison

To demonstrate the significant advantages of our proposed methodology, we applied a weight vector *w* = (0.3,05,0.2)^*T*^ and compared it to previous techniques [33–42]. The results for various techniques on the data provided in Table 3 are presented in Table 3. This table confirms that the suggested approach identifies the best alternative, providing evidence of the effectiveness and rationality of the innovative strategy. In addition, it is evident that the proposed methodology offers novel concepts and ideas that differ from existing techniques. Prior techniques mainly utilized basic measurements and distance measures to summarize alternatives in the form of IVPFSs and IVIFSs. In contrast, our methodology not only analyzes ideal values ℋ^−^ and ℋ^+^ but also evaluates the relative relevance of weighted distance measures. Furthermore, the proposed technique efficiently handles DM processes with interval weights data, a capability lacking in existing methods. Moreover, it is important to note that various techniques classified as IFSs PFSs, IVPFSs, IVIFSs, and CIFSs are considered special cases of the suggested methodology. Therefore, the proposed approach has a broader scope of application and provides a more comprehensive solution for DM processes than existing methods.

**Fig 3:**
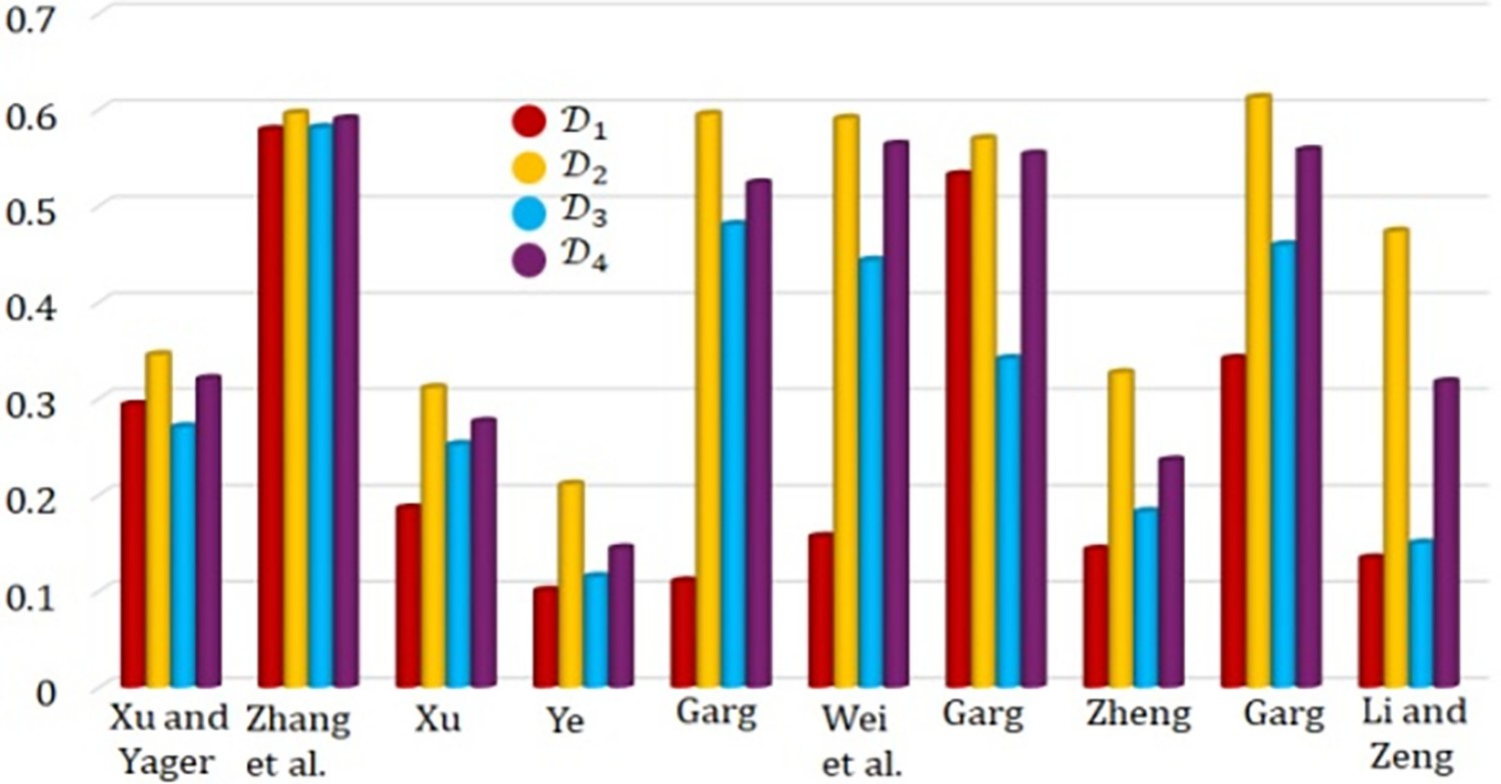
Score values and ranking order of alternative with different approaches.

Fig 3 presents a visual representation of the data displayed in Table 7. The weight vector for each alternative in relation to every criterion is computed using the entropy measure as defined in reference [2]. This measure considers the level of uncertainty or variability in the data, allowing for a more accurate and reliable determination of the weights. By using the entropy measure, we can assess the relative importance of each criterion and ensure that the weights assigned to each alternative are reflective of their true significance in the decision-making process.

**Table 7:** Comparative ranking of different approaches.

Approaches	Rating values				Ranking order
	$D_{1}$	$D_{2}$	$D_{3}$	$D_{4}$	
Xu and Yager [40]	0.2934	0.3452	0.2704	0.3203	$D_{2}≻D_{4}≻D_{3}≻D_{1}$
Zhang et al. [41]	0.5788	0.5958	0.5810	0.5899	$D_{2}≻D_{4}≻D_{3}≻D_{1}$
Xu [42]	0.1860	0.3110	0.2520	0.2758	$D_{2}≻D_{4}≻D_{3}≻D_{1}$
Ye [43]	0.1005	0.2108	0.1149	0.1445	$D_{2}≻D_{4}≻D_{3}≻D_{1}$
Garg [44]	0.1103	0.5948	0.4804	0.5235	$D_{2}≻D_{4}≻D_{3}≻D_{1}$
Wei et al. [45]	0.1564	0.5906	0.4431	0.5636	$D_{2}≻D_{4}≻D_{3}≻D_{1}$
Garg [46]	0.5321	0.5695	0.3411	0.5534	$D_{2}≻D_{4}≻D_{3}≻D_{1}$
Zheng [47]	0.1433	0.3265	0.1824	0.2357	$D_{2}≻D_{4}≻D_{3}≻D_{1}$
Garg [48]	0.3413	0.6123	0.4592	0.5578	$D_{2}≻D_{4}≻D_{3}≻D_{1}$
Li and Zeng [49]	0.1341	0.4733	0.1497	0.3171	$D_{2}≻D_{4}≻D_{3}≻D_{1}$

### 6.1. Advantages of the proposed method

The NLP models that have been developed so far, including the NLP model under CIFS suggested by Garg and Kaur [50], the NLP model under IVIFS presented by Zeng et al [51], and the NLP model under IFS proposed by Li [12], are inadequate for handling the proposed case study due to their limitations. These models cannot effectively cope with scenarios where $U^{+}+V^{+}≼1$, which is a significant drawback.

The NLP method proposed in this study provides decision-makers with a flexible framework to evaluate alternatives based on their preferences. The method operates within a cubic Pythagorean fuzzy environment, which allows decision-makers to express their uncertainty and vagueness in the evaluation process. One of the key advantages of the proposed method is its ability to consider multiple types of fuzzy sets, including PFSs, IVPFSs, and CPFSs, simultaneously. This approach is more efficient in handling uncertainty in the DM problem compared to existing methods, which typically consider only one type of fuzzy set. By considering multiple types of fuzzy sets, the proposed method can provide decision-makers with more accurate and reliable outcomes. Moreover, the outcomes of the proposed method are not only more accurate but also more practical and reasonable. The method considers the practical constraints and limitations that decision-makers face in the real world. This consideration makes the method more suitable for real-world decision-making problems. Thus, the proposed NLP method is a flexible and efficient approach for handling uncertainty in the DM problem. By simultaneously considering PFSs, IVPFSs, and CPFSs, the method provides decision-makers with more accurate and reliable outcomes. The method is also practical and reasonable, making it suitable for real-world decision-making problems.

### 6.2 Limitations

1. It is essential to recognize and address the limitations of this study, as with any research. Although this study collected data from decision-makers on three criteria and four alternatives, there is still scope for improvement in future research by including a more extensive range of attributes and alternatives. Therefore, it is crucial to consider these limitations and conduct further research to enhance understanding of the subject.
2. It is worth noting that in certain real-life scenarios, the condition ${\left(U^{+}\right)}^{2}+{\left(V^{+}\right)}^{2}≼1$ may not hold true. For instance, when the provided rating values about the object is (〈[0.7,0,8],[0.8,0.9]〉,〈0.6,0.4〉), the is clear that the sum 0.8^2^ and 0.9^2^ exceeds 1, and this makes it impossible to apply the proposed method. Such situations pose a significant challenge in decision-making, and alternative methods need to be explored to address them effectively. It is important to conduct further research to develop new techniques that can handle scenarios where the condition ${\left(U^{+}\right)}^{2}+{\left(V^{+}\right)}^{2}≼1$is not met, thus improving the reliability and accuracy of decision-making processes in practical applications.

## 7. Conclusion

This paper presents an innovative method for multiple-attribute decision-making (MADM) that leverages CPFSs alongside NLP techniques and the TOPSIS approach. The proposed method seeks to enhance the precision and efficiency of decision-making processes in complex situations involving multiple attributes and conflicting goals. By integrating these techniques, the approach effectively addresses uncertainty and vagueness in decision-making, leading to more dependable and precise outcomes. To illustrate the efficacy of the proposed NLP model, we provide a practical example of a GSS. This case study demonstrates how the method can successfully manage multiple attributes and conflicting objectives, resulting in improved decision-making. The GSS case study underscores the advantages of the method and shows its capability to navigate complex decision-making scenarios. Additionally, by comparing the outcomes of the proposed method with those of several existing approaches, we have validated its accuracy and stability. Finally, we discuss the strengths and limitations of the proposed algorithm, with the intention of applying it to other uncertain environments in future research [52–56].

## Supporting information

S1 File (DOCX)
