# Supplementary material for: Enhancing green supplier selection: A nonlinear programming method with TOPSIS in cubic Pythagorean fuzzy contexts
Source: PLoS One. 2024 Dec 5;19(12):e0310956. doi: 10.1371/journal.pone.0310956 (PMC11620431; doi:10.1371/journal.pone.0310956)
Supplement: S1 File — (DOCX) [file pone.0310956.s001.docx]

The MATLAB coding for model as presented in Equation (12) as follows:

n = 5; %example value and can be change according to the situation,

% Initialize the vectors for w, v, p, q, U_ij, U_ij_star, V_ij, and ξ_ij

w = rand(1, n);

v = rand(1, n);

p = rand(1, n);

q = rand(1, n);

U_ij = rand(1, n);

U_ij_star = rand(1, n);

V_ij = rand(1, n);

xi_ij = rand(1, n);

num = 0;

for j = 1:n

num = num + sqrt(w(j) * (1 - U_ij_star(j)^2)) + v(j) * (V_ij(j)^2) + p(j) * (U_ij(j))^2 + sqrt(q(j) * (1 - V_ij(j)^2));

end

num = sqrt(num);

% Calculate the denominator

denom_1 = 0;

denom_2 = 0;

for j = 1:n

denom_1 = denom_1 + sqrt(w(j) * (1 - U_ij_star(j)^2)) + v(j) * (V_ij(j)^2) + p(j) * (xi_ij(j))^2 + sqrt(q(j) * (1 - V_ij(j)^2));

denom_2 = denom_2 + sqrt(w(j) * (1 - U_ij_star(j)^2)) + v(j) * (V_ij(j)^2) + p(j) * (U_ij(j))^2 + sqrt(q(j) * (1 - V_ij(j)^2));

end

denom_1 = sqrt(denom_1);

denom_2 = sqrt(denom_2);

% Calculate C_i

C_i = num / (denom_1 + denom_2);

% Display the result

disp(['C_i = ', num2str(C_i)]);

**Explanation:**

1. **Variables**:
   - w, v, p, q are vectors that contain the weights for each term in the summation.
   - U_ij, U_ij_star, V_ij, and xi_ij are the variables used in the formula, and they are also vectors.
   - n is the number of terms in the summation.
2. **Summation**:
   - The numerator is calculated by summing the components as per the equation, then taking the square root of the sum.
   - The denominator is computed in two parts, denom_1 and denom_2, which are also summed and then square rooted.
3. **Final Calculation**:
   - C_i is calculated by dividing the numerator by the sum of the two parts of the denominator.

The MATLAB coding for model as presented in Equation (25) as follows:

n = 5; %example value and can be change according to the situation,

% Initialize the vectors for w, v, p, q, U_ij_minus, V_ij_plus, V_ij

w = rand(1, n); % example values

v = rand(1, n); % example values

p = rand(1, n); % example values

q = rand(1, n); % example values

U_ij_minus = rand(1, n); % example values

V_ij_plus = rand(1, n); % example values

V_ij = rand(1, n); % example values

% Calculate the numerator

numerator = 0;

for j = 1:n

numerator = numerator + (w(j) * U_ij_minus(j)) + sqrt(v(j) * (1 - (V_ij_plus(j))^2)) + sqrt(p(j) * (1 - (V_ij(j))^2)) + (q(j) * V_ij(j));

end

% Calculate the denominator

denominator = 0;

for j = 1:n

denominator = denominator + (w(j) + v(j) + p(j) + q(j));

end

% Calculate C_i^-

C_i_minus = min(numerator / denominator);

% Display the result

disp(['C_i^- = ', num2str(C_i_minus)]);

The MATLAB coding for model as presented in Equation (27) as follows:

n = 5; %example value and can be change according to the situation,

n = 5; % example value, you can change it

% Initialize the vectors for w, U_ij_minus, V_ij_plus, U_ij, and V_ij

w = rand(1, n); % example values

U_ij_minus = rand(1, n); % example values

V_ij_plus = rand(1, n); % example values

U_ij = rand(1, n); % example values

V_ij = rand(1, n); % example values

% Calculate the summation part of the equation

summation = 0;

for j = 1:n

summation = summation + w(j) * (U_ij_minus(j) + sqrt(1 - V_ij_plus(j)) + sqrt(1 - U_ij(j)) + V_ij(j));

end

% Calculate C_i^-

C_i_minus = (1/4) * summation;

% Display the result

disp(['C_i^- = ', num2str(C_i_minus)]);

% Define the value of m

m = 5; % example value, you can change it

% Initialize the vector p^ik (p_ik)

p_ik = rand(1, m); % example values

% Calculate the summation part of the equation

summation = 0;

for k = 1:m

summation = summation + (p_ik(k) + m/2 - 1);

end

% Calculate D_i

D_i = 1 / (m * (m - 1)) * summation;

% Display the result

disp(['D_i = ', num2str(D_i)]);

the MATLAB code to calculate Di (Equation 18)​ based on the equation you provided:

matlab

Copy code

% Define the value of m

m = 5; % example value, you can change it

% Initialize the vector p^ik (p_ik)

p_ik = rand(1, m); % example values

% Calculate the summation part of the equation

summation = 0;

for k = 1:m

summation = summation + (p_ik(k) + m/2 - 1);

end

% Calculate D_i

D_i = 1 / (m * (m - 1)) * summation;

% Display the result

disp(['D_i = ', num2str(D_i)]);

**Explanation:**

1. **Variables**:
   - m is the value specified in the equation (the number of terms).
   - p_ik is a vector representing the values of pikp^ikpik (denoted as p_ik in MATLAB).
2. **Summation**:
   - The summation is calculated by adding pik+m2−1p^ik + \frac{m}{2} - 1pik+2m​−1 for each k from 1 to m.
3. **Final Calculation**:
   - DiD_iDi​ is calculated by multiplying the sum by 1m(m−1)\frac{1}{m(m-1)}m(m−1)1​.
4. **Output**:
   - The result is displayed as DiD_iDi​.

can adjust the values of m and p_ik to fit your specific needs.
